# Supplementary material for: A unified framework for estimating country-specific cumulative incidence for 18 diseases stratified by polygenic risk
Source: Nat Commun. 2024 Jun 12;15:5007. doi: 10.1038/s41467-024-48938-2 (PMC11169548; doi:10.1038/s41467-024-48938-2)
Supplement: Supplementary file 3 — Description of Additional Supplementary Files [file 41467_2024_48938_MOESM3_ESM.pdf]

Descriptions of Additional Supplementary Files

File Name: Supplementary Data 1.
Description: Descriptive statistics (prevalence, follow-up and age at onset) for each phenotype by study.

File Name: Supplementary Data 2.
Description: Meta-analyzed Hazard Ratios per Standard Deviation of a disease relevant polygenic risk score. Age, sex and 'age and sex' stratified results also provided. The p-value is from a Wald test and HetPval is from Cochran’s Q Test from the rma function in the metafor package.

File Name: Supplementary Data 3.
Description: Heterogeneity test for each age quartile for both sexes combined as well as stratified by sex for meta-analyzed hazard ratios. The heterogeneity p-value is from Cochran’s Q Test.

File Name: Supplementary Data 4.
Description: Country-specific cumulative absolute risk for PGS strata across 19 phenotypes.

File Name: Supplementary Data 5.
Description: Country and sex-specific age at attainment of clinical risk threshold for Type 2 Diabetes

File Name: Supplementary Data 6.
Description: Country-specific age at attainment of clinical risk threshold for Breast Cancer.

File Name: Supplementary Data 7.
Description: Comparison of country-specific cumulative incidence when using biobank specific hazard ratios and meta-analysed hazard ratios in participants of European ancestries.

File Name: Supplementary Data 8.
Description: Harmonized definitions of flagship diseases using ICD-10 and ICD-9 codes.

File Name: Supplementary Data 9.
Description: Phenotype matching between Global Burden of Disease and UK Biobank

File Name: Supplementary Data 10.
Description: Genome-wide association study summary statistics used to compute PGS and sample overlap with each biobank.

File Name: Supplementary Data 11.
Description: Age of onset quartiles by study.

File Name: Supplementary Data 12.
Description: Phenotype definitions utilising primary care data from the UK Biobank
