# Supplementary material for: A unified framework for estimating country-specific cumulative incidence for 18 diseases stratified by polygenic risk
Source: Nat Commun. 2024 Jun 12;15:5007. doi: 10.1038/s41467-024-48938-2 (PMC11169548; doi:10.1038/s41467-024-48938-2)
Supplement: Supplementary file 16 — Reporting Summary [file 41467_2024_48938_MOESM16_ESM.pdf]

nature portfolio

Corresponding author(s): Samuli RIpatti

Last updated by author(s): April 1, 2024

Reporting Summary

Nature Portfolio wishes to improve the reproducibility of the work that we publish. This form provides structure for consistency and transparency in reporting. For further information on Nature Portfolio policies, see our Editorial Policies and the Editorial Policy Checklist.

Statistics

For all statistical analyses, confirm that the following items are present in the figure legend, table legend, main text, or Methods section.

n/a	Confirmed	
☐	☒	The exact sample size (*n*) for each experimental group/condition, given as a discrete number and unit of measurement
☒	☐	A statement on whether measurements were taken from distinct samples or whether the same sample was measured repeatedly
☐	☒	The statistical test(s) used AND whether they are one- or two-sided<br>*Only common tests should be described solely by name; describe more complex techniques in the Methods section.*
☐	☒	A description of all covariates tested
☐	☒	A description of any assumptions or corrections, such as tests of normality and adjustment for multiple comparisons
☐	☒	A full description of the statistical parameters including central tendency (e.g. means) or other basic estimates (e.g. regression coefficient) AND variation (e.g. standard deviation) or associated estimates of uncertainty (e.g. confidence intervals)
☐	☒	For null hypothesis testing, the test statistic (e.g. *F*, *t*, *r*) with confidence intervals, effect sizes, degrees of freedom and *P* value noted<br>*Give P values as exact values whenever suitable.*
☒	☐	For Bayesian analysis, information on the choice of priors and Markov chain Monte Carlo settings
☒	☐	For hierarchical and complex designs, identification of the appropriate level for tests and full reporting of outcomes
☒	☐	Estimates of effect sizes (e.g. Cohen's *d*, Pearson's *r*), indicating how they were calculated

Our web collection on statistics for biologists contains articles on many of the points above.

Software and code

Policy information about availability of computer code

Data collection	No software was used for data collection.
Data analysis	Custom scripts used to estimate baseline cumulative incidences from the Global Burden of disease and in each biobank—define phenotypes, calculate hazard ratios with Cox proportional hazards models, meta-analyze hazard ratios, and estimate cumulative incidences—are in a GitHub repository: https://github.com/intervene-EU-H2020/flagship<br>Each biobank undertakes its own genotyping, imputation and quality control using a combination of the following tools, with more information provided in the Supplementary Methods Plink 1.9 and 2.0, Ensembl VEP, IMPUTE4, Eagle 2.x, Minimac3 v2.0.1, GenomeStudio, Beagle 5.1, GenoPred, bcftools, vcftools, Shapeit2.<br>MegaPRS was used to calculate polygenic scores using published GWAS summary statistics.<br>For data wrangling, visualization and plotting of results, we used R version 3.x.x, specifically packages ggplot, tidyverse, survminer, survival, metafor, tidyverse, data.table.<br>Slightly different version numbers for software listed were used across biobanks depending on computational environment.

For manuscripts utilizing custom algorithms or software that are central to the research but not yet described in published literature, software must be made available to editors and reviewers. We strongly encourage code deposition in a community repository (e.g. GitHub). See the Nature Portfolio guidelines for submitting code & software for further information.

Data

Policy information about availability of data

All manuscripts must include a data availability statement. This statement should provide the following information, where applicable:

- Accession codes, unique identifiers, or web links for publicly available datasets
- A description of any restrictions on data availability
- For clinical datasets or third party data, please ensure that the statement adheres to our policy

Individual level data in this study are not publicly available due to legal and privacy limitations, but they can be accessed through individual participating biobanks. The FinnGen data may be accessed through Finnish Biobanks' FinBB portal (www.finbb.fi; email: info.fingenious@finbb.fi). The Trøndelag Health Study (HUNT). The HUNT data may be accessed by application to the HUNT Research Centre. Estonian Biobank. Researchers interested in Estonian Biobank can request the access at https://www.geenivaramu.ee/en/access-biobank. De-identified data of the MGB Biobank that supports this study is available from the MGB Biobank portal. Restrictions apply to the availability of these data, which are available to MGB-affiliated researchers via a formal application. UK Biobank data are available through a procedure described at http://www.ukbiobank.ac.uk/using-the-resource/. Genomics England data is available through an application process described here: https://www.genomicsengland.co.uk/research/academic/join-gecip. Generation Scotland data may be accessed through an application process described here: https://www.ed.ac.uk/generation-scotland/for-researchers/access

Summary level data are reported in tables and supplementary tables. The GWAS summary statistics used are publicly available and listed here: http://ftp.ebi.ac.uk/pub/databases/gwas/summary_statistics/GCST004001-GCST005000/GCST004988/

http://diagram-consortium.org/downloads.html

http://ftp.ebi.ac.uk/pub/databases/gwas/summary_statistics/GCST006001-GCST007000/GCST006085/

http://www.cardiogramplusc4d.org/data-downloads/

http://ftp.ebi.ac.uk/pub/databases/gwas/summary_statistics/GCST90011001-GCST90012000/GCST90011809/

http://ftp.ebi.ac.uk/pub/databases/gwas/summary_statistics/GCST010001-GCST011000/GCST010043/

http://ftp.ebi.ac.uk/pub/databases/gwas/summary_statistics/GCST90013001-GCST90014000/GCST90013445/

http://ftp.ebi.ac.uk/pub/databases/gwas/summary_statistics/GCST006001-GCST007000/GCST006061/

https://figshare.com/articles/dataset/mdd2018/14672085

http://ftp.ebi.ac.uk/pub/databases/gwas/summary_statistics/GCST90018001-GCST90019000/GCST90018875/

http://ftp.ebi.ac.uk/pub/databases/gwas/summary_statistics/GCST90013001-GCST90014000/GCST90013534/

http://ftp.ebi.ac.uk/pub/databases/gwas/summary_statistics/GCST007001-GCST008000/GCST007091/

http://ftp.ebi.ac.uk/pub/databases/gwas/summary_statistics/GCST007001-GCST008000/GCST007090/

http://ftp.ebi.ac.uk/pub/databases/gwas/summary_statistics/GCST008001-GCST009000/GCST008972/

http://ftp.ebi.ac.uk/pub/databases/gwas/summary_statistics/GCST007001-GCST008000/GCST007343/

https://www.ebi.ac.uk/gwas/studies/GCST90044141

he Global Burden of Disease data used here is available from https://vizhub.healthdata.org/gbd-results/

Research involving human participants, their data, or biological material

Policy information about studies with human participants or human data. See also policy information about sex, gender (identity/presentation), and sexual orientation and race, ethnicity and racism.

Reporting on sex and gender

We performed analyses in each biobank on the full sample and in a sex-stratified manner. We discuss in the methods the heuristic for using sex-specific models for a given phenotype. In most biobanks, sex is identified through genotyping identification of XX or XY chromosomes.

Reporting on race, ethnicity, or other socially relevant groupings

This study uses individuals of European ancestry as determined by genetically estimated ancestry. Each biobank may define this slightly differently, but usually by clustering principal components of genotypes using 1000G reference. For UK Biobank, Ancestry assignment uses methodology and scripts from GenoPred (https://opain.github.io/GenoPred/DiverseAncestry.html). Individuals were stratified into one of five super populations African (AFR), American (AMR), South Asian (SAS), East Asian (EAS) and European (EUR). The 1000 Genomes data acted as a reference given the individuals are known to belong to one of the 5 super populations. More information on each biobank is in the Supplementary Material under Ancestry Assignment.

Population characteristics

This study included participants from different study cohorts, all of European ancestry (typically estimated by principal component analysis from genotyping compared to 1000G European population). The key characteristics of the participants in the cohorts are described in the main text, Supplementary text, Supplementary Tables 5 and 7.

Recruitment

This study included participants from different study cohorts with either population-based, hospital-based, or mixed recruitment designs. For example, FinnGen is a random sample of subjects from Finnish population-based and clinical biobanks. A proportion of FinnGen was ascertained through hospital biobanks and disease-based collections, but the the hazard ratios for PRS categories and disease prevalences were similar between FinnGen and the population-based FINRISK cohort. HUNT is a The periodic population-based health survey design includes three recruitment waves—HUNT1 (1984-1986), HUNT2 (1995-1997), and HUNT3 (2006-2008)—concentrated in the North-Trøndelag area, where all adults > 20 years of age were invited to participate. Participations rates across waves vary but are greater than 50%. UKB is also a population-based study but with lower participation rates, there has been demonstrated healthy participation bias. To address this, we use data from the Global Burden of Disease 2019 Study to estimate baseline hazards per country/state rather than using each study cohort with its potential ascertainment biases.

Ethics oversight

Patients and control subjects in FinnGen provided informed consent for biobank research, based on the Finnish Biobank Act. Alternatively, separate research cohorts, collected prior the Finnish Biobank Act came into effect (in September 2013) and start of FinnGen (August 2017), were collected based on study-specific consents and later transferred to the Finnish biobanks after approval by Fimea (Finnish Medicines Agency), the National Supervisory Authority for Welfare and Health. Recruitment

protocols followed the biobank protocols approved by Fimea. The Coordinating Ethics Committee of the Hospital District of Helsinki and Uusimaa (HUS) statement number for the FinnGen study is Nr HUS/990/2017.
The FinnGen study is approved by Finnish Institute for Health and Welfare (permit numbers: THL/2031/6.02.00/2017, THL/1101/5.05.00/2017, THL/341/6.02.00/2018, THL/2222/6.02.00/2018, THL/283/6.02.00/2019, THL/1721/5.05.00/2019 and THL/1524/5.05.00/2020), Digital and population data service agency (permit numbers: VRK43431/2017-3, VRK/6909/2018-3, VRK/4415/2019-3), the Social Insurance Institution (permit numbers: KELA 58/522/2017, KELA 131/522/2018, KELA 70/522/2019, KELA 98/522/2019, KELA 134/522/2019, KELA 138/522/2019, KELA 2/522/2020, KELA 16/522/2020), Findata permit numbers THL/2364/14.02/2020, THL/4055/14.06.00/2020, THL/3433/14.06.00/2020, THL/4432/14.06/2020, THL/5189/14.06/2020, THL/5894/14.06.00/2020, THL/6619/14.06.00/2020, THL/209/14.06.00/2021, THL/688/14.06.00/2021, THL/1284/14.06.00/2021, THL/1965/14.06.00/2021, THL/5546/14.02.00/2020, THL/2658/14.06.00/2021, THL/4235/14.06.00/2021, Statistics Finland (permit numbers: TK-53-1041-17 and TK/143/07.03.00/2020 (earlier TK-53-90-20) TK/1735/07.03.00/2021, TK/3112/07.03.00/2021) and Finnish Registry for Kidney Diseases permission/extract from the meeting minutes on 4th July 2019.
The Biobank Access Decisions for FinnGen samples and data utilized in FinnGen Data Freeze 10 include: THL Biobank BB2017_55, BB2017_111, BB2018_19, BB_2018_34, BB_2018_67, BB2018_71, BB2019_7, BB2019_8, BB2019_26, BB2020_1, BB2021_65, Finnish Red Cross Blood Service Biobank 7.12.2017, Helsinki Biobank HUS/359/2017, HUS/248/2020, HUS/150/2022 § 12, §13, §14, §15, §16, §17, §18, and §23, Auria Biobank AB17-5154 and amendment #1 (August 17 2020) and amendments BB_2021-0140, BB_2021-0156 (August 26 2021, Feb 2 2022), BB_2021-0169, BB_2021-0179, BB_2021-0161, AB20-5926 and amendment #1 (April 23 2020)and it´s modification (Sep 22 2021), Biobank Borealis of Northern Finland_2017_1013, 2021_5010, 2021_5018, 2021_5015, 2021_5023, 2021_5017, 2022_6001, Biobank of Eastern Finland 1186/2018 and amendment 22 § /2020, 53§/2021, 13§/2022, 14§/2022, 15§/2022, Finnish Clinical Biobank Tampere MH0004 and amendments (21.02.2020 & 06.10.2020), §8/2021, §9/2022, §10/2022, §12/2022, §20/2022, §21/2022, §22/2022, §23/2022, Central Finland Biobank 1-2017, and Terveystalo Biobank STB 2018001 and amendment 25th Aug 2020, Finnish Hematological Registry and Clinical Biobank decision 18th June 2021, Arctic biobank P0844: ARC_2021_1001.
Ethics approval for the UK Biobank study was obtained from the North West Centre for Research Ethics Committee (11/NW/0382). UK Biobank data used in this study were obtained under approved application 78537.
The genotyping in Trøndelag Health Study and work presented in here was approved by the Regional Committee for Ethics in Medical Research, Central Norway (2014/144, 2018/1622, 2018/411492). All participants signed informed consent for participation and the use of data in research.

Ethical approval for the GS:SFHS study was obtained from the Tayside Committee on Medical Research Ethics (on behalf of the National Health Service)
The activities of the EstBB are regulated by the Human Genes Research Act, which was adopted in 2000 specifically for the operations of the EstBB. Individual level data analysis in the EstBB was carried out under ethical approval 1.1-12/624 from the Estonian Committee on Bioethics and Human Research (Estonian Ministry of Social Affairs), using data according to release application S22, document number 6-7/GI/16259 from the Estonian Biobank.

The informed consent process for the Genomics England 100,000 Genomes Project has been approved by the National Research Ethics Service Research Ethics Committee for East of England—Cambridge South Research Ethics Committee.

The analysis using Mass General Brigham Biobank is approved under IRB protocol 2022P001736.

Note that full information on the approval of the study protocol must also be provided in the manuscript.

Field-specific reporting

Please select the one below that is the best fit for your research. If you are not sure, read the appropriate sections before making your selection.

☒ Life sciences ☐ Behavioural & social sciences ☐ Ecological, evolutionary & environmental sciences

For a reference copy of the document with all sections, see nature.com/documents/nr-reporting-summary-flat.pdf

Life sciences study design

All studies must disclose on these points even when the disclosure is negative.

Sample size	Sample size per disease per biobank is in Supplementary Data 1. The study makes use of 7 studies in 4 countries (N=1,197,129). No power calculation was done because we used all genotyped samples with paired phenotype data in the biobanks participating in the consortia. Because the standardized PGS was significantly associated with each outcome in each biobank, we believe our results are well powered for stratification across age, sex, and PGS strata. The sample size for GWAS used for the polygenic risk scores are described in Supplementary Data 10 and statistically well powered.
Data exclusions	Exclusion of samples and variants was based on standard guidelines and quality control procedures and were pre-established by each biobank as described in Supplementary Methods. Briefly, each biobank performed genetic ancestry assignment to stratify individuals into geographic super populations and individuals not falling into the European population were excluded. Genotyped samples without paired phenotype information were excluded. Samples with poor genotyping quality were excluded with slightly different criteria within each biobank. For FinnGen, samples were removed if ● Pihat was > 0.9 and the samples were not monozygotic or replicates<br>● There was a discrepancy between reported sex and genetically determined sex (F-value ≤ 0.3 for females and ≥ 0.8 for males)<br>● Missingness was ≥ 5%<br>● Heterozygosity was ±4 standard deviations from the population average<br>● Pihat was > 0.1 with 14 or more samples<br>● Samples were ±4 standard deviations away from the population average according to the first two genetic principal components.

Replication	For country specific cumulative incidences per a given disease, we made use of all the biobank data currently available. Replication of these cumulative incidences can be done in additional biobanks or biobank recruitment waves in the future.
Randomization	The study does not include allocation of participants to different experimental groups.
Blinding	No blinding was applied in this observational study.

Reporting for specific materials, systems and methods

We require information from authors about some types of materials, experimental systems and methods used in many studies. Here, indicate whether each material, system or method listed is relevant to your study. If you are not sure if a list item applies to your research, read the appropriate section before selecting a response.

Materials & experimental systems

n/a	Involved in the study
☒	☐ Antibodies
☒	☐ Eukaryotic cell lines
☒	☐ Palaeontology and archaeology
☒	☐ Animals and other organisms
☒	☐ Clinical data
☒	☐ Dual use research of concern
☒	☐ Plants

Methods

n/a	Involved in the study
☒	☐ ChIP-seq
☒	☐ Flow cytometry
☒	☐ MRI-based neuroimaging

Plants

Seed stocks	*Report on the source of all seed stocks or other plant material used. If applicable, state the seed stock centre and catalogue number. If plant specimens were collected from the field, describe the collection location, date and sampling procedures.*
Novel plant genotypes	*Describe the methods by which all novel plant genotypes were produced. This includes those generated by transgenic approaches, gene editing, chemical/radiation-based mutagenesis and hybridization. For transgenic lines, describe the transformation method, the number of independent lines analyzed and the generation upon which experiments were performed. For gene-edited lines, describe the editor used, the endogenous sequence targeted for editing, the targeting guide RNA sequence (if applicable) and how the editor was applied.*
Authentication	*Describe any authentication procedures for each seed stock used or novel genotype generated. Describe any experiments used to assess the effect of a mutation and, where applicable, how potential secondary effects (e.g. second site T-DNA insertions, mosiacism, off-target gene editing) were examined.*
